# Randomized clinical trial testing the efficacy and safety of 0.5% colchicine cream versus photodynamic therapy with methyl aminolevulinate in the treatment of skin field cancerization: study protocol

**DOI:** 10.1186/s12885-018-4288-7

**Published:** 2018-03-27

**Authors:** Anna Carolina Miola, Eliane Roio Ferreira, Luciana Patricia Fernandes Abbade, Juliano Vilaverde Schmitt, Helio Amante Miot

**Affiliations:** Department of Dermatology and Radiotherapy, Botucatu Medical School, Universidade Estadual Paulista, UNESP, São Paulo, Brazil

**Keywords:** Actinic keratoses, Colchicine, Methyl aminolevulinate, Skin cancer, Skin field cancerization, Photodynamic therapy

## Abstract

**Background:**

The primary clinical manifestation of skin field cancerization is the presence of actinic keratoses (AKs). Current treatments for AKs related to skin field cancerization include photodynamic therapy (PDT) and colchicine. The objective of this study is to evaluate the efficacy and safety of 0.5% colchicine cream versus PDT with methyl aminolevulinate (MAL-PDT) in the treatment of skin field cancerization.

**Methods:**

In a randomized controlled and open clinical trial with a blind histopathological and immunohistochemical analysis, 36 patients with up to 10 AKs on their forearms will be included from the outpatient clinic. The forearms will be randomized into two groups, clinically evaluated and biopsied for histopathology and immunohistochemistry (p53 and Ki67). One forearm will be treated with 0.5% colchicine cream for 10 days, and the other forearm will receive one session of MAL-PDT; the forearms will subsequently be reassessed clinically and histologically after 60 days (T60) of treatment. The primary endpoint will be the point of complete clearance of AKs in T60. The sample size will enable a detection in the reduction of over 10% in AK counts between the groups with power of 0.9 and an alpha of 0.05, accounting for an estimated dropout rate of 10%, resulting in 36 patients (72 forearms). All participants included in the randomized study will be part of the analysis, and the final outcomes of any dropouts will be the value of their last visit (LOCF). The statistical analysis will be performed using SPSS 22.0, and a *p* value < 5% will be considered to be significant.

**Discussion:**

It is expected that colchicine will be superior to MAL-PDT in reducing AKs and in the skin field cancerization, and there will be good tolerability in both groups. Colchicine intervention is novel in that it provides a new alternative to MAL-PDT. Moreover, this drug is inexpensive that may be a potential treatment of skin field cancerization that can be prescribed in public health systems with good results.

**Trial registration:**

The trial is registered in Brazilian Registry for Clinical Trials (Registration number: RBR-8y3sj9, date assigned May 4, 2016, retrospectively registered).

## Background

### Actinic keratosis

Actinic keratosis (AK), the most common premalignant lesion, affects sun-exposed areas as a result of chronic exposure to UVR [1]. Individuals with skin types I and II of the Fitzpatrick classification with excessive sun exposure, advanced age or immunosuppression comprise the highest risk group [2].

AK lesions may be macular or papular injuries with adherent and rough scales, a color varying from yellow to brown, and a size of approximately 0.5 to 1 cm, and they can converge on plaques. AKs occur primarily in the areas most exposed to sunlight, such as the face, scalp, forearms and backs of hands [3].

Infiltration, rapid growth, bleeding and ulceration may indicate the progression of AK to squamous cell carcinoma (SCC) [4], the incidence of which varies between 0.025% and 20% of patients with AKs per year [5]. AKs are the most obvious clinical manifestation of skin field cancerization activity and are used to quantify this activity due to the absence of other quantification methods.

### Field cancerization

First described in 1953 by Slaughter, field cancerization refers to an apparently normal tegument area with subclinical and multifocal changes, composed of genetically altered cells [6]. The concept of skin field cancerization suggests that the apparently normal skin adjacent to actinic keratoses (AKs) is the basis for the clonal expansion of genetically altered cells. This phenomenon can explain the appearance of new AKs or other nonmelanoma skin tumors in the same area, as well as the local recurrence of tumors considered to be completely excised by histopathological analysis [7].

Recently, skin field cancerization has been extensively studied, due to its clinical importance. The alteration of adjacent cells makes it necessary to treat the area as a whole [8] to prevent recurrence, the appearance of new lesions and the progress of existing lesions.

Treatment modalities that aim to stabilize the activity of skin field cancerization are warranted because they may decrease morbidity and mortality in the exposed population and prevent future neoplastic and preneoplastic injuries [9].

### Available treatments

The purpose of treating AKs and skin field cancerization is to mitigate the risk of the AKs progressing to malignant lesions and cancer development [10]. Several treatment modalities exist, which are chosen according to the following: size of the area, number of lesions, immunosuppression or other co-morbidities, cost of treatment, tolerability and patient adherence [11].

Cryotherapy with liquid nitrogen, which is the most common AK treatment, is well tolerated, easily accessible and shows good clinical and histopathological results (clinical response of 83% with a freezing time longer than 20 s) [12]. Discomfort during the application and post-procedure hypochromia can occur and may persist [13]. Cryotherapy treats only visible lesions, making it a good treatment for AKs, but this therapy does not treat skin field cancerization [14].

Another treatment option is 5-fluorouracil (5FU), a topical chemotherapy and antimetabolic drug that disrupts DNA synthesis by thymidylate synthase binding [15]. At concentrations ranging from 0.5 to 5%, 5FU has been shown to produce good therapeutic responses in treating AK, but aggressive side effects can occur, such as erythema, pain, swelling and ulceration [16]. 5FU improves the overall appearance of skin, decreases p53 expression and stabilizes skin field cancerization even after six months of treatment [17].

Imiquimod is a topical immunomodulator *toll-like receptor 7* agonist [18], which stimulates local immunity with consequent apoptosis by injury. Imiquimod is commercially available in 3.75% and 5% formulations. Unlike 5-FU, which may cause erythema, edema and local variable discomfort, treatment with 5% imiquimod, applied three times per week for eight weeks, has been shown to cause an 82% reduction of AKs with only slight moderate local irritation reported. [19]

Ingenol mebutate is a *Euphorphia peplus*-derived ester with two distinct forms of action: either direct cell death induction through changes in the mitochondrial and plasma membranes of dysplastic cells or local immune response induced by proinflammatory cytokines and massive neutrophil infiltration [20]. The application of 0.05% ingenol mebutate twice a day for two consecutive days showed a complete response rate of 71%. Moreover, with minimal systemic absorption, ingenol mebutate has tolerable side effects, such as erythema, crusting and pain [21].

Diclofenac is a non-steroidal anti-inflammatory drug that inhibits the enzyme cyclooxygenase type 2, the activity of which is increased in AKs and non-melanoma skin tumors [22]. When compared to a placebo, topical diclofenac at 3% in 2.5% hyaluronic acid gel used for 90 days demonstrated an efficacy of 50% compared with 3% in the placebo group. Adverse events, such as erythema, pruritus, contact dermatitis and skin xerosis, are rare; however, Diclofenac requires a long treatment period for an adequate response [23].

In addition to the abovementioned treatments, other treatments for skin field cancerization are known, such as resiquimod, tretinoin [24], colchicine and photodynamic therapy (PDT); the latter two are included in this study protocol.

### Photodynamic therapy

Photodynamic therapy (PDT) is a treatment modality that induces cytotoxicity of the proliferating cells of a tumor through a light emission source; this activity is localized through a photosensitizing agent which can be oral or topical [25].

In dermatology, PDT is used for the treatment of pre-malignant and non-melanoma skin tumors, such as AKs, superficial basal cell carcinoma and Bowen disease, or SCC in situ [26]. PDT is also used for skin rejuvenation and to treat inflammatory skin diseases, such as psoriasis, warts, morphea and Kaposi’s sarcoma [27].

For the implementation of PDT, patients should first be exposed to a photosensitizing agent, usually a topical one. The following two photosensitizing agents are licensed: 5-aminolevulinic acid (ALA-PDT) and its lipophilic derivative, methyl aminolevulinate (MAL) [28]. MAL is preferred for its non-saturable absorption mechanism, which results in greater penetration and thus greater uptake by neoplastic cells [29].

MAL is a precursor of protoporphyrin IX, which generates reactive oxygen in dysplastic keratinocytes when exposed to a light source with the appropriate wavelength [30].

MAL is topically administered in a 16% cream formulation and maintained under occlusion for 3 h before the light source exposure, which can originate from one of three sources: a broad-spectrum lamp, a light-emitting diode lamp (LED) or a laser [31].

During PDT, MAL is activated and generates reactive oxygen. During this process, MAL is converted from its resting state to an active form called *singlet*, which has a short half-life. Due to the presence of *singlet*, cells start to undergo changes in their membranes, which affect the permeability and transport of substances and cause toxicity of cytoplasmic organelles, inducing apoptosis in cancer cells [32].

PDT has shown response rates close to 90% in thin and moderate AKs on the face and scalp, and that response has been shown to be sustained for one year in 39 to 63% of patients with only one session and 78% of patients with two sessions. In addition, a good tolerability, minimal adverse effects with only a short duration, and very satisfactory aesthetic results have been shown [33].

### Colchicine

Colchicine is an antimitotic agent that binds to microtubular proteins and interferes with the mitotic spindle activity, interrupting mitosis at metaphase [34, 35]. Colchicine also inhibits the chemotaxis of polymorphonuclear leukocytes. Side effects can include abdominal pain, nausea, vomiting, agranulocytosis and aplastic anemia. Topical application can cause erythema, edema, crusts and vesicles in the treated area. Toxicity is serious and can occur with any route of administration. Colchicine has a slow elimination, preferably through the intestine and kidney [36].

In dermatology, colchicine has several therapeutic applications, both systemic and topical, such as treating the following: psoriasis vulgaris, generalized pustular psoriasis, pustulosis palmar plantar, small vessel vasculitis, type II reaction of leprosy, Behcet’s disease and others [37].

Topical colchicine was first used for treating AKs in 1968 by Marshall [38], and few studies on colchicine have been conducted since.

Colchicine has been studied at concentrations of 0.5 and 1% in a gel and has been demonstrated to reduce AKs by 77% with satisfactory after-treatment aesthetic results [39].

However, there have been few studies that have systematized the dosage and duration of treatment with colchicine, and no studies comparing the efficacy of colchicine and photodynamic therapy with methyl aminolevulinate (MAL), a treatment modality that has been tested in more clinical trials for skin field cancerization. Therefore, the present study was undertaken.

### Objectives

The objective of this study is to evaluate the efficacy and safety of 0.5% colchicine cream versus PDT with methyl aminolevulinate (MAL-PDT) in treating skin field cancerization in patients with multiple actinic keratoses.

### Research question and hypotheses

Is 0.5% colchicine cream more effective than MAL-PDT treatment for the complete clearance of AKs (primary endpoint), partial clearance of AKs, histological modification of the atypical profile, p53 and Ki67 expression and tolerability (secondary outcomes) after 60 days of treatment?

Main hypotheses are as follows:

1. Colchicine and MAL-PDT (as proposed schemes) will both reduce the number of AKs and modify skin field cancerization histology.
2. Colchicine and MAL-PDT (proposed schemes) will both show good tolerability and safety in skin field cancerization treatment.
3. Colchicine will be superior to MAL- PDT (proposed schemes) in AK reduction and in modifying skin field cancerization histology.

## Methods/Design

This randomized clinical trial will be controlled and open with blind histopathological and immunohistochemical analyses. Thirty-six patients with 3 to 10 AKs on each forearm will be treated with colchicine cream on one forearm and MAL-PDT on the other forearm. Patients will be evaluated for the number of AKs and the expression of Ki67 and p53 before and after the treatments.

This study was approved by the Medical Ethics Committee of the institution and recorded in REBEC under RBR-8y3sj9.

### Selection of participants

Inclusion criteria

- Age over 18 years for both sexes
- Participants who present three to ten lesions clinically compatible with AKs on each forearm, bilaterally, who have not undergone treatment for AKs or field cancerization in the last six months

Exclusion criteria

- Number of lesions is less than three or greater than ten on each forearm
- Selected treatment area has atypical clinical appearance or other extensive skin disease affecting the forearms
- Current clinical diagnosis or evidence of any medical condition that expose the patient to increased risk or interferes with the safety or efficacy of the proposed treatment
- Presence of hypersensitivity or allergy to any of the substances under study
- Patients using any topical or systemic immunosuppressive substance, oral retinoid or other local treatments (corticosteroids, anti-inflammatories, retinoids)
- Immunocompromised individuals
- Coagulation disorders
- Pregnancy suspected or confirmed
- Women of childbearing potential not using contraception
- Women who are breast-feeding

Discontinuation criteria

- Withdrawal of consent
- Presence of infection (erysipelas, cellulitis or abscess) in the monitored treatment areas. In such cases, research subject should be properly treated as a routine service
- Patients using methods of treatment different than those proposed in this study
- Serious adverse events, according to investigator
- Pregnancy during follow-up

### Participant recruitment

Thirty-six patients from the dermatology department of the Botucatu Medical School will be recruited. Patients will be informed regarding the possibility of participating in this processing study during their dermatological consultation. All of the patients will sign a consent form before enrollment, and they will be scheduled for outpatient treatment for the study itself without loss of follow-up after study participation.

### Randomization and concealment of allocation of treatments

Patient forearms will be randomized by computer simulation (block randomization) for treatment with 0.5% colchicine cream or MAL-PDT. Inclusion will be held consecutively to the scheduling of patients, regardless of the randomization list.

### Interventions

The study units of analysis are forearms, the treatment of which will be randomized to groups designated as PDT-MAL and COL.

In the COL group, patients will be recommended to use 0.5% cream twice daily for 10 days on the forearm. Adherence to the treatment will be monitored by phone calls. In the MAL-PDT group, forearms will receive one MAL-PDT session [40], administered by the same researcher. The protocol for the MAL-PDT session is as follows: AK lesions will first be curetted. Next, a layer of 16% methyl aminolevulinate will be applied, and the forearm will be occluded for 3 h while covered by PVC film and aluminum foil. Next, the forearm will be illuminated with a light emitting diode (LED) with a wavelength of 630 nm at a distance of five to eight centimeters for eight minutes. All patients will wear safety glasses to protect their eyes.

The study will last 60 days for each patient. During this time, patients will be instructed to apply SPF 30 broad spectrum sunscreen (UVA and UVB) to their forearms.

The area of AK count, evaluation and punch 3.0 mm biopsy will be standardized. The assessed area will have a distal limit as a line at the metacarpophalangeal joints and a proximal limit as a line extending from antecubital fossa to the lateral epicondyle of the anterior side of the forearms. Biopsies will be taken in central region of the middle third of each assessed area.

**Fig. 1:**
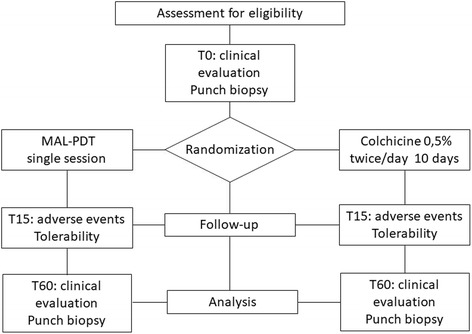
Flow chart of the study (CONSORT)

Participants will be assessed at the following times: T0 - inclusion, randomization, clinical evaluation (AK count), skin biopsy (bilateral) and beginning of treatment; T15 - assessment of adverse effects and tolerability; T60 - clinical evaluation (AK count), reassessment of adverse effects and tolerability, and bilateral skin biopsy [see Fig. 1].

### Endpoints

Primary endpoint:

- Complete *clearance* rate of AKs when treated with colchicine and MAL-PDT in T60 secondary outcomes
- Partial *clearance* rate of AKs when treated with colchicine and MAL-PDT, comparing T0 to T60
- Modification of histological atypia profile between treatments, comparing T0 to T60
- Modification of the expression of p53 and Ki67 between treatments, comparing T0 to T60
- Adverse effects related to the proposed treatment: impact of local events and severity
- Emergence of non-melanoma skin tumors
- Tolerability assessed by the patient reporting adverse effects, preferably between treatments.

### Data analysis

The main study endpoints will be the longitudinal evaluation of AK count, histological changes and proliferation of scores (p53 and Ki67).

Diagnosis of AKs will be performed based on a clinical examination by a certified dermatologist. Counts of AKs will be performed twice by same researcher after the lesions are marked with a felt-tip pen, with no distinction being made between visible and palpable forms [41].

Epithelial atypia will be evaluated (T0 and T60) based on H&E staining according to KIN classification (*Keratinocyte Intraepithelial Neoplasia*) in three central fields in interfollicular areas, and the investigator will not know which group the samples are from.

For immunohistochemistry, histological sections of 4 μm thickness will be mounted on silanized slides and subjected to immunohistochemical staining for detection of Ki67 (Cell Sig. Tech., Inc., Danvers, MA, USA, mouse mAb IgG1, #9449) or p53 (Cell Sig. Tech., Inc., Danvers, MA, USA, mouse mAb Ig2b, #48818). After sections deparaffinization and rehydration will be performed the endogenous peroxidase blockade with 3% H_2_O_2_, followed by heat-induced antigen retrieval and protein blockade by treatment with milk. The sections will be incubated overnight at 4 °C with the primary antibody diluted 1:300 or 1:150 for the Ki67 and p53 respectly. Signal amplification will be performed by biotin-free polymer detection system using the MACH 4® universal HRP-Polymer kit (Biocare Medical, Pike Lane Concord, CA, USA) according to the manufacturer’s instructions. Lastly, 3–3′ diaminobenzedine will be used as a chromogenic substrate and the sections will be counterstained with Harris hematoxylin. Epithelial expression of p53 and Ki67 will be evaluated (T0 and T60) based upon the HSCORE, calculated in three central fields (40×), of the sampled epithelium, and the investigator will not know from which group the samples are obtained.

Patients will be questioned at T15 and T60 regarding whether they would accept this treatment again and which treatment they preferred. Any AKs remaining after the study will be treated with cryotherapy with liquid nitrogen.

### Sample size

The sample size was chosen to detect a reduction in AKs of over 10% between groups and a standard deviation of equivalent differences. A power of 0.9 and an alpha of 0.05 were adopted, and the dropout rate was estimated to be 10%, resulting in 36 patients (72 forearms).

### Statistical analysis

All participants included in study will be randomized in the ITT (intention to treat) group. Data analysis will be performed for the ITT population following the generalized linear mixed-effects model. The final outcomes for dropouts will be the value of last visit (LOCF).

Regarding the group descriptions, categorical variables will be presented as the absolute number and percentage. Continuous variables will be assessed for normality using the Kolmogorov-Smirnov test (Lilliefors) and represented by means and standard deviations or medians and quartiles (p25-p75).

The AK count and the analysis of the histopathological data will be compared with respect to time and group (over time) using the linear mixed-effects model with robust covariance matrix and probability adjustment for each distribution.

Data will be tabulated in Microsoft Excel 2013, and statistical analyses will be performed using SPSS 22.0; a *p* value < 5% will be considered to be significant.

## Discussion

Dissemination of the results of this study should reach the scientific community through conferences and scientific publications, as well as the institutional research and ethics committee where the work was developed.

Results of this study may help to reduce the costs of skin field cancerization treatment and expand new treatment strategies. The primary limitation of this trial is the impossibility of clinical blinding; however, the histopathological and immunohistochemical analyses will be blind, and the AK count prior to treatment will also be blind, thereby reducing biases.

Based upon the results of this study, dermatologists may approach new treatments, and skin field cancerization treatment may lead to a lower incidence of keratinocytic cancer, thereby directly benefiting society.

## Abbreviations

5-FU: 5-fluorouracil
AK: Actinic keratosis
ALA: 5-aminolevulinic acid
LED: Light emitter diode
MAL: Methyl-aminolevulinate
MAL: With methyl-aminolevulinate
PDT: Photodynamic therapy
PDT: Photodynamic therapy
R: Radiation
UV: Ultraviolet
UV: Ultraviolet
